# Getting Up to Speed: Rapid Pathogen and Antimicrobial Resistance Diagnostics in Sepsis

**DOI:** 10.3390/microorganisms12091824

**Published:** 2024-09-03

**Authors:** Mariana P. Liborio, Patrick N. A. Harris, Chitra Ravi, Adam D. Irwin

**Affiliations:** 1 UQ Centre for Clinical Research, The University of Queensland, Herston, QLD 4029, Australia; m.pitombeiraliborio@uq.edu.au (M.P.L.); c.ravi@uq.edu.au (C.R.); 2 Herston Infectious Disease Institute, Metro North, QLD Health, Herston, QLD 4029, Australia; 3 Central Microbiology, Pathology Queensland, Royal Brisbane and Women’s Hospital, Herston, QLD 4006, Australia; 4 Infection Management and Prevention Service, Queensland Children’s Hospital, Brisbane, QLD 4101, Australia

**Keywords:** rapid diagnostics, bloodstream infection, sepsis diagnosis, pathogen diagnosis, antimicrobial resistance

## Abstract

Sepsis is a life-threatening organ dysfunction caused by a dysregulated host response to infection. Time to receive effective therapy is a primary determinant of mortality in patients with sepsis. Blood culture is the reference standard for the microbiological diagnosis of bloodstream infections, despite its low sensitivity and prolonged time to receive a pathogen detection. In recent years, rapid tests for pathogen identification, antimicrobial susceptibility, and sepsis identification have emerged, both culture-based and culture-independent methods. This rapid narrative review presents currently commercially available approved diagnostic molecular technologies in bloodstream infections, including their clinical performance and impact on patient outcome, when available. Peer-reviewed publications relevant to the topic were searched through PubMed, and manufacturer websites of commercially available assays identified were also consulted as further sources of information. We have reviewed data about the following technologies for pathogen identification: fluorescence in situ hybridization with peptide nucleic acid probes (Accelerate Pheno^TM^), microarray-based assay (Verigene^®^), multiplex polymerase chain reaction (cobas^®^ eplex, BioFire^®^ FilmArray^®^, Molecular Mouse, Unyvero BCU System^TM^), matrix-assisted laser desorption-ionization time-of-flight mass spectrometry (Rapid MBT Sepsityper^®^), T2 magnetic resonance (T2Bacteria Panel), and metagenomics-based assays (Karius^©^, DISQVER^®^, Day Zero Diagnostics). Technologies for antimicrobial susceptibility testing included the following: Alfed 60 AST^TM^, VITEK^®^ REVEAL^TM^, dRAST^TM^, ASTar^®^, Fastinov^®^, QuickMIC^®^, Resistell^TM^, and LifeScale. Characteristics, microbiological performance, and issues of each method are described, as well as their clinical performance, when available.

## 1. Introduction

Sepsis is defined as a life-threatening organ dysfunction brought on by a dysregulated host response to infection [1] and is a priority in global health [2]. A major factor determining mortality in patients with sepsis is the time to receive effective antibiotic therapy [3]. Unfortunately, empiric antibiotic therapy may be ineffective in up to 40% of patients because of the diversity of pathogens and prevalence of antimicrobial resistance (AMR) mechanisms [4,5]. The empiric use of broad-spectrum antibiotics contributes to the increase and further spread of AMR [6,7] and leads to complications, adverse drug reactions, and *Clostridioides difficile* toxin-related diseases [8].

Blood culture (BC) is the existing reference standard for the microbiological diagnosis of bloodstream infections (BSIs) [9]. The diagnostic limitations and uncertainties of blood cultures relate to a low sensitivity, and prolonged time to receive pathogen detection and subsequent antimicrobial susceptibility reporting. These issues lead to the liberal use of broad-spectrum antimicrobial therapy [10]. Exposure to antimicrobials prior to BC sample collection further reduces the sensitivity of the test [11]. Being on antibiotic therapy is an independent factor for lower pathogen identification in blood cultures of sepsis patients, leading to as low as 27% of pathogen identification in this scenario [12]. More highly sensitive tests than BC are required to exclude the presence of infection and identify when antibiotics are not required [13].

Novel diagnostic test providing rapid pathogen and antimicrobial susceptibility identification results in BSI have emerged, both culture-based and culture-independent methods. Although there is no universal consensus on the definition of ‘rapid’, it has been proposed to define it as obtaining the result within a working day shift (i.e., ~8 h) [14].

**Figure 1:**
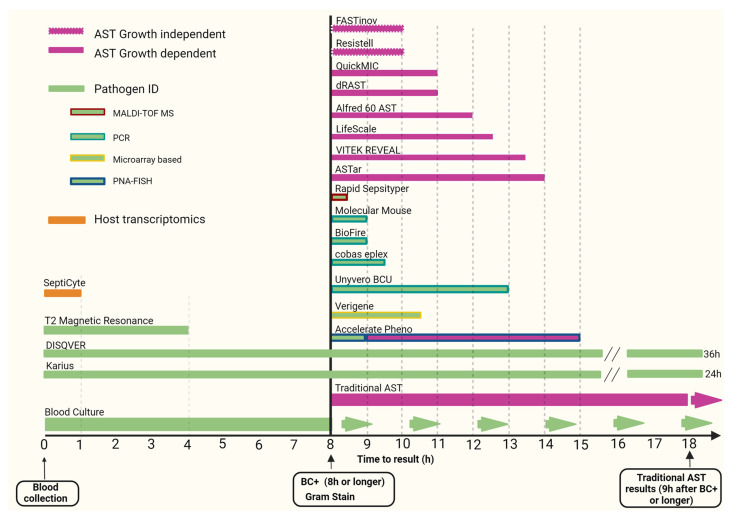
Time to receive results of selected rapid diagnostic tests in sepsis. Abbreviations: ID—identification; MALDI-TOF MS—matrix-assisted laser desorption-ionization time-of-flight mass spectrometry; PNA-FISH—fluorescence in situ hybridization with peptide nucleic acid probes; PCR—polymerase chain reaction; AST—antimicrobial susceptibility testing; BC+—blood culture positivity. Day zero diagnostics does not specify how many hours are needed for results.

This rapid review provides an update and assessment of improvements in pathogen and antimicrobial resistance detection in sepsis over the last decade, focusing primarily on commercially available approved diagnostic molecular technologies (see Figure 1).

## 2. Materials and Methods

We performed a rapid narrative review to appraise peer-reviewed publications on rapid pathogen diagnostics over the last decade (2014–2024). The following search strategy was applied to PubMed (last accessed July 2024):

((Polymerase Chain Reaction[MH] OR “Polymerase Chain Reaction”[tiab] OR metagenomic*[tiab] OR Molecular Diagnostic Techniques[MeSH Terms] OR “AMR”[tiab] OR “antimicrobial resistance”[tiab] OR “Drug Resistance, Bacterial”[Mesh]) AND (rapid[tiab] OR fast[tiab])) OR (“blood culture”[MeSH Terms] OR “blood culture”[Title/Abstract] OR whole blood[tiab]) AND (“pathogen detection”[Title/Abstract]) OR “microbial identification”[Title/Abstract]) OR (ACCELERATE PHENO[tiab] OR VERIGENE[tiab] OR EPLEX BCID[tiab] OR BIOFIRE BLOOD[tiab] OR T2 MAGNETIC RESONANCE[tiab] OR KARIUS[tiab] OR DISQVER [tiab] OR Alfred 60[tiab] OR Alifax[tiab] OR VITEK[tiab] OR dRAST[tiab] OR ASTar[tiab] OR Fastinov[tiab] OR Septicyte[tiab]) AND (“Sepsis”[Mesh] OR “Bacteremia”[Mesh] OR Sepsis[tiab] OR Septicemia[tiab] OR Septicaemia[tiab] OR Bacteremia[tiab] OR Bacteraemia[tiab] OR “blood stream infection*”[tiab]). Filters: from 2014 to 2024.

Papers *(n* = 1303) identified through this search, including original articles, reviews, and case reports, were revised to identify CE-marked or FDA approved commercial assays for rapid pathogen identification (targets for gram-positive and gram-negative bacteria), rapid antimicrobial susceptibility testing, and complementary approaches including host transcriptomics for sepsis identification, from whole blood and blood culture, whose performance had been assessed in peer-reviewed studies. Papers describing in-house or non-commercial technologies were excluded from further consideration. 

Screening included title and abstract and when the information could not be found, also full texts. This screening enabled the identification of the following currently available commercial technologies for pathogen identification of bloodstream infections from whole blood and blood culture:

Accelerate Pheno (Accelerate Diagnostics, Inc., Tucson, AZ, USA)
Verigene (Nanosphere, Northbrook, IL, USA)
ePlex BCID (Roche Diagnostics, Rotkreuz, Switzerland)
Biofire Blood Culture Identification (bioMérieux SA, Marcy-l’Etoile, France)
T2 Magnetic Resonance (T2 Biosystems, Lexington, MA, USA)
Karius (Karius, Redwood City, California)
DISQVER (Noscendo GmbH, Duisburg, Germany)

This screening enabled the identification of the following currently available commercial technologies for rapid antimicrobial susceptibility testing of bloodstream infections from whole blood and blood culture:

Alfred 60/AST (Alifax^®^, Polverara, Italy)
VITEK^®^ REVEAL^TM^ (bioMérieux SA, Marcy-l’Etoile, France) (Formerly Specific Reveal)
dRAST (QuantaMatrix, Seoul, Republic of Korea)
ASTar (Q-linea AB, Uppsala, Sweden)
Fastinov (Fastinov, Matosinhos, Portugal)

This screening enabled the identification of the following currently available commercial technology for host transcriptomics in bloodstream infections:

Septicyte (Immunexpress, Seattle, WA, USA)

The following other technologies were also identified but not included because they were discontinued, not used for bloodstream infections, or no recent studies evaluating their performance were found:

Acuitas (OpGen, Rockville, MD, USA) (discontinued)
SepsiTest (Molzym, Bremen, Germany) (no studies after 2016)
Micro-Dx (Molzym, Bremen, Germany) (no studies with blood)
Hybcell Pathogen DNA assay (Cube Dx, St. Valentin, Austria) (1 study from2016)
iDTECT Dx Blood test (PathoQuest, Paris, France) (discontinued)
Magicplex (Seegene, Seoul, Republic of Korea) (discontinued)
Lightcycler SeptiFast (Roche Diagnostics, Rotkreuz, Switzerland) (discontinued)
VYOO (SIRS-Lab, Jena, Germany) (discontinued)
IRIDICA system (Abbott, Chicago, IL, USA) (discontinued)
iCubate (iCubate, Huntsville, AL, USA) (no study after 2019)
PNA FISH ADVANDX (OpGen, Rockville, MD, USA) (no studies after 2020)

The reference lists of the identified studies were searched to identify any additional relevant material. 

Manufacturer websites of commercially available assays identified through PubMed were also consulted as further sources of information. 

## 3. Blood Culture-Dependent Methods

Culture-dependent methods for pathogen identification and antimicrobial susceptibility testing are currently the most established. They have been extensively evaluated over the years, are cheap, generally easy to perform, and available in all clinical microbiology laboratories. The incubation phase, inherent within a culture-based method, provides a high quantity of organisms for subsequent testing. Despite this, the limitations of culture include the following: the time required for the growth of each pathogen, specific cultivation techniques requirements using different incubation conditions for some organisms, and the existence of medically relevant organisms that do not grow in cultures. As a result, the sensitivity of blood culture systems to detect pathogens may be reduced [15]. 

### 3.1. Fluorescence In Situ Hybridization with Peptide Nucleic Acid Probes (PNA-FISH)

#### Accelerate Pheno^TM^

The Accelerate Pheno^TM^ system (Accelerate Diagnostics, Inc., Tucson, AZ, USA) uses gel electrofiltration and fluorescence in situ hybridization with peptide nucleic acid probes (PNA-FISH) technology for pathogen identification (ID) in combination with automated microscopic imaging for analysing bacterial growth rates and for extrapolating minimum inhibitory concentration (MIC) values for antibiotic susceptibility testing (AST).

The current Accelerate Pheno^TM^ system ID panel for positive blood culture bottles (BCBs) covers a range of two yeasts (*Candida albicans* and *Candida glabatra*), six gram-positive bacterial species, eight gram-negative bacterial species, consisting of six Enterobacterales (*Escherichia coli*, *Klebsiella* spp., *Enterobacter* spp., *Serratia marcescens*, *Proteus* spp., *Citrobacter* spp.), and two non-fermenting pathogens (*Pseudomonas aeruginosa* and *Acinetobacter baumannii*) (Table 1). It is an easy-to-use system that requires no specific technical knowledge and less than 5 min of hands-on time. The BCB supernatant is transferred to a vial that is introduced into the device, with the subsequent analysis of ID and AST fully automated [16].

Fluorescence in situ hybridization or FISH is an established technique to detect and localize specific sequences of nucleic acids. The Accelerate Pheno^TM^ technology enables rapid hybridization of several mono-labelled DNA oligonucleotides that target ribosomal RNA, involving an array of simultaneous FISH tests. For each test, a signal from a target probe is compared with a signal from universal bacterial and eukaryotic probes. Using digital microscopy, colocalization of the target probe signal with a universal probe signal confirms the presence and identity of the target while differentiating from non-specific staining [17]. AST is performed by morphokinetic cellular analysis, which tracks phenotypic features including the size, shape, and division rate of individual live cells growing into micro-colonies while being challenged by specific antibiotics concentrations using automated microscopy and time-lapse imaging, and the MIC results are provided [18]. 

Its software has undergone improvements over time, with common blood culture contaminant species such as *Staphylococcus warneri* and *Streptococcus oralis* added to the group of detectable coagulase-negative staphylococci (CoNS) and *Streptococcus* species, respectively. *Streptococcus pneumoniae* was removed from the panel but instead reported on the genus level. In addition, software adjustments were made to minimize non-specific and indeterminate detections. The system is not able to perform the identification of off-panel organisms but can detect and correctly report their presence in a few cases [19].

The ID and AST results are obtained within 1 and 7 h, respectively, directly from positive blood culture bottles (BCBs), and in practical scenarios time to receive results have been considerably reduced compared to standard methods, such as MALDI-TOF MS (Microflex LT; Bruker Daltonics, Ettlingen, Germany), with average of 45 to 90 min for ID [20] and Vitek 2 (bioMérieux SA, Marcy-l’Étoile, France) and disk diffusion for AST, with results in 2 to 18 h and 18 to 48 h, respectively [21,22,23]. The system also allows for a reduction in hands-on time compared to conventional methods [24].

The clinical performance of this test was extensively evaluated since 2017, when it was approved by U.S. Food and Drug Administration (FDA). The overall pathogen identification agreement between the Accelerate Pheno^TM^ system and conventional culture methods varied from 96.2% to 100% in monomicrobial cultures across different studies, with lower agreement results when polymicrobial cultures were analysed (40% to 100%) [22,25,26]. 

The overall categorical agreement (CA) between the Accelerate Pheno^TM^ system and culture-based AST varies from 93.7% to 96.4% [20,21,23], with one study finding decreased CA for beta-lactams (cefepime 84.4%, piperacillin-tazobactam 86.5%, ceftazidime 87.6%) and for *Pseudomonas aeruginosa* (71.9%; with cefepime 33.3%, piperacillin-tazobactam 77.8%, ceftazidime 0%) [22]. Additional testing is needed for some organisms (i.e., *Acinetobacter baumannii* and carbapenemase-producing gram-negative organisms) and for *Streptococcus* spp., for which the antimicrobials tested and interpretations reported can vary greatly depending on the species that are present [24].

In a multicentre study involving 13 clinical sites in the USA, the Accelerate Pheno^TM^ system accurately identified 14 common bacterial pathogens and two *Candida* spp. with sensitivities ranging from 94.6 to 100%. In regard to AST, for gram-positives, the overall essential agreement (EA) and CA were 97.6% and 97.9%, respectively. Overall, very major error (VME), major error (ME), and minor error (mE) rates were 1.0%, 0.7%, and 1.3%, respectively, when compared to broth microdilution or disk diffusion. For gram-negatives, overall EA and CA were 95.4% and 94.3%, respectively. Overall VME, ME, and mE rates were 0.5%, 0.9%, and 4.8%, respectively [27].

Improvements in the analysis of polymicrobial samples and in AST algorithms, notably beta-lactam testing in both *Pseudomonas aeruginosa* and Enterobacterales, are required for their implementation in routine workflows [22]. Performance analysis using the latest versions of the blood culture kit and of the system (version 1.4) appears to show more reliable susceptibility testing results for Enterobacterales for most of the commonly used antimicrobial agents [28]. 

In bacteraemic patients, the implementation of the Accelerate Pheno^TM^ is associated with reduced lengths of stay (median 6.3 vs. 8.1 days), achievement of optimal therapy (93.6% vs. 84.6%), time to receive optimal therapy (1.4 vs. 2.4 days), and total antimicrobial days of therapy (6 vs. 7 days) when integrated into a healthcare system with an active antimicrobial stewardship program [29]. In a 2020 randomized trial by Banerjee et al. evaluating the clinical impact of the system in gram-negative bacteraemia, it also led to faster changes in antibiotic therapy, although there were no differences in patient outcomes between the arms randomized to standard of care (SOC) testing with antimicrobial stewardship (AMS) review or Accelerate Pheno^TM^ system with AMS [30]. A third study described a decreased median time to receive antibiotic susceptibility testing (29.4 h decrease), decreased median time to receive preferred antimicrobial therapy (21.2 h decrease), decreased utilization of broad-spectrum agents (days of therapy [DOTs]/1000 days present—PRE 655.2 vs. POST 585.8), and increased use of narrow spectrum beta-lactams (69.1 vs. 141.7), though there were no difference in clinical outcomes for patients with gram-negative bloodstream infections [31]. 

### 3.2. Microarray-Based Assay

#### Verigene^®^

Verigene^®^ gram-positive blood culture (BC-GP) and gram-negative blood culture (BC-GN) nucleic acid tests (Nanosphere, Northbrook, IL, USA) are microarray-based assays capable of testing multiple bacterial pathogens and their antibiotic resistances simultaneously, from positive blood cultures, within 2.5 h and with hands-on time of 5 min. The gram-positive assay consists of thirteen bacterial targets (including *Staphylococcus*, *Streptococcus*, *Enterococcus*, *Listeria*) and three resistance markers (*mecA*, *vanA*, and *vanB*). The gram-negative blood culture kit consists of nine bacterial targets (although it cannot distinguish *Escherichia coli* from *Shigella* sspspp.) and six resistance markers, including the most prevalent extended spectrum beta-lactamase (ESBL) (e.g., CTX-M) and common carbapenemases (IMP, KPC, NDM, OXA-48-like, VIM) [32]. Following a positive blood culture, gram staining is needed to decide which panel to use. 

The gram-positive assay can correctly identify organisms in more than 89.6% of positive monomicrobial blood cultures, but this number drops to 62.5% in polymicrobial cultures [33]. Similar findings, with better identification rates for monomicrobial cultures, were described in other studies [34,35]. For gram-negatives, the system correctly identifies pathogens from 80.5 to 93.8% of monomicrobial blood culture bottles, and 77.8% in polymicrobial cultures [36,37]. Polymicrobial sepsis is associated with worse patient outcomes than monomicrobial sepsis. The current prevalence of polymicrobial sepsis is estimated to be up to 14%, although the true burden is likely underestimated [38].

Resistance gene *mecA* can be detected by Verigene^®^ in over 95.2% organisms where it is present, with slightly better detection rates in *Staphylococcus aureus* than in *Staphylococcus epidermidis*, suggesting that de-escalation of vancomycin to a narrower anti-staphylococcal agent could be performed based on the BC-GP assay result [34]. With the ever-evolving transference of resistance mechanisms between bacteria, it is important to know that the system reports the presence of *mecA* for *S. aureus* and *S. epidermidis* only and not in other species [35]. Although it can detect three resistance markers for gram-positives and six for gram-negatives, resistances caused by other mechanisms are not detectable by the Verigene^®^ system.

The greatest advantage of the Verigene^®^ system is the earlier time for organism identification and susceptibility results when compared to standard culture-based methods [39]. Pathogen identification and antimicrobial susceptibility results can be reported to the treating physician the same day that blood cultures are taken from patients [40].

A study by Walker et al. in 2016 was already able to demonstrate that length of intensive care unit (ICU) stays (12 vs. 16.2 days), 30-day mortality (8.1% vs. 19.2%), and mortality associated with multidrug-resistant organisms (12.5% vs. 63%) were significantly lower in patients with gram-negative bacteraemia after the Verigene^®^ BC-GN was implemented [41]. In a 2017 prospective study in Japan evaluating the impact of the Verigene^®^ system on antibiotic prescriptions in patients with community-onset bacteraemia, 27.6% (of 185 cases) had antibiotic prescription changes within 2 days after Verigene^®^ analysis, including bacteraemia due to gram-negatives and gram-positives [42]. 

### 3.3. Multiplex Polymerase Chain Reaction

#### 3.3.1. Cobas^®^ Eplex System (Formerly ePlex^®^ System) [43]

The Cobas^®^ Eplex (Roche Diagnostics, Rotkreuz, Switzerland) blood culture identification (BCID) panels are fully automated polymerase chain reaction (PCR)-based assays designed to identify 20 g-positive bacterial genera or species and four antimicrobial resistance genes (BCID-GP panel), 21 g-negative bacterial genera or species and six antimicrobial resistance genes (BCID-GN panel), and 16 fungal genera or species (BCID-FP panel) (Table 1), within 1.5 h from a positive blood culture. They are the broadest multiplex PCR panels available for the rapid diagnosis of bloodstream infections (BSIs), which leads to potentially improved antimicrobial therapy decisions [44]. They include the detection of potential blood culture contaminants and antimicrobial resistance gene mecC (rare cause of methicillin-resistant *Staphylococus aureus* [MRSA]). Non-specialized personnel can perform the test [45].

The gram-positive panel has Pan gram-negative and Pan Candida targets, which are unique features of the panel that alert the user to the presence of mixed cultures that may not be detected by the gram stain [46], and assist in situations where gram stains are misinterpreted and can help identify additional species that may be present in cases of gram variability [47]. 

In the same way, the gram-negative panel has Pan gram-positive and Pan Candida targets. In the absence of a Pan target, choosing the appropriate test panel becomes more dependent on accurately interpreting the primary gram stain result, which may lead to the identification of an off-panel organism or an undetected mixed infection. [8]. 

It is worth mentioning the inclusion of *Staphylococcus lugdunensis* in the ePlex^®^ panel, as it is a common pathogen with species specific virulence factors [48]; the inclusion of pathogens with resistance to many antibiotics, such as *Stenotrophomonas maltophilia*, can facilitate early initiation of trimethoprim-sulfamethoxazole; and anaerobic gram-negative rods, such as *Fusobacterium* spp. or *Bacteroides fragilis* in the BCID-GN [49], prompting the addition of anaerobic coverage to empirical antimicrobial regimens.

The ePlex^®^ BCID-GP Panel distinguishes between *E. faecalis* and *E. faecium* species, allowing an earlier switch toward beta-lactam antibiotics if *E. faecalis* is identified. It also identifies gram-positive rods belonging to the genera Corynebacterium, Lactobacillus, Bacillus (B. subtilis group and B. cereus group), and the anaerobic species *Cutibacterium acnes*, allowing for quicker de-escalation of antibiotic treatment in the event that a contaminant is found [46].

Several studies have evaluated the clinical performance of the Eplex^®^ system. The positive percent agreement (PPA) showed by the BCID-GN was 97.2% in a study by McCarty et al. in the USA, similar to the 96% PPA reported by both Huang et al. in Belgium and Bryant et al. in France and exceeds the 89.7% PPA observed by Krifors et al. in Sweden and the 92.9% observed in a U.S. multicentre trial by Wolk et al. [8,46,50,51,52].

For the BCID-GP panel, sensitivity was 94.7% by Oberhettinger et al. in Germany, overall agreement was 96% in a study by Briant et al. in France, positive percentage agreement 100% by McCarty et al. [48,51,53]. Lastly, in the U.S., it was reported by Carol et al. that the overall sample accuracy was above 89% [47].

The ePlex^®^ system performs better than other commercial broad-range multiplex PCR systems for polymicrobial samples, but most of its discrepancies are still in polymicrobial samples [51], with achievement of accurate identification of all pathogens ranging from 88% to 100% in these samples [46,48], if they are covered by the panel.

Also, unique to this panel are the Pan targets, present in the Pan-GN and Pan-GP assays. They can detect isolates growing organisms not visualized by microscopy and will alert the laboratory of a possible second organism that might be significant in triggering further diagnostic procedures, carrying out broad-based empirical treatment, or introducing antifungal therapy [47].

The clinical impact of the ePlex^®^ system is reflected in the significant reduction in time to receive optimal antimicrobial therapy (43.4 with ePlex vs. 72.1 h with standard of care) [54] and early antimicrobial optimization, including discontinuation of unnecessary antibiotics [50] or antimicrobial escalation if resistance genes or organisms with intrinsic resistance are detected [55]. A recent randomized controlled trial showed optimized antimicrobial treatment 12 h after transmission of BC positivity and gram stain results from the first positive BC (90/105 = 85.7%, CI95% [77.5; 91.8] vs. 68/107 = 63.6%, CI95% [53.7; 72.6]; *p* < 0.01), leading to early termination of the study after the inclusion of 309 patients [56].

#### 3.3.2. BioFire^®^ Blood Culture Identification

BioFire^®^ FilmArray^®^ blood culture ID (BCID) system is a two-stage, highly multiplexed, nested PCR test that is carried out in a closed, disposable, single-use pouch. It requires about 2 min for assay setup and provides results in approximately 1 h. It is designed to identify simultaneously 24 etiologic agents of sepsis (8 g-positive, 11 g-negative, and 5 Candida species) as well as three antimicrobial resistance genes (*mecA*, *vanA/B*, and KPC).

The sensitivity of the method is over 97.3% for species identification, with specificity above 99%. The sensitivity and specificity for *vanA/B* and KPC are 100%; those for *mecA* are 98.4% and 98.3%, respectively [57]. It demonstrated a much shorter turnaround time compared to standardised culture-based methods (2.4 vs. 26.5 h) [58] with clinical utility in the adjustment of empiric antimicrobial therapy in the critically ill septic patient [59].

The newest version of the BCID (BioFire^®^ blood culture identification 2 panel, BCID2, bioMérieux) was released in 2021, encompassing 43 molecular targets associated with BSI, including 11 g-positive bacteria, 15 g-negative bacteria, 7 yeast species, and 10 resistance markers. The last targets include genes encoding carbapenemases (IMP, KPC, OXA-48-like, NDM, and VIM), colistin resistance (*mcr-1*), extended spectrum beta-lactamase (ESBL) (CTX-M), methicillin resistance (*mecA/C*) and, specifically for MRSA, *mecA/C* and MREJ [mec right-extremity junction]), or vancomycin resistance (*vanA/B*) [60].

The ability to distinguish between *E. faecalis* and *E. faecium* was incorporated into the previous version, which was a significant improvement. When combined with the capacity to detect *vanA-vanB*, this ability could be helpful in antibiotic stewardship program (ASP) interventions, enabling the reduction in empirical vancomycin therapies for *E. faecalis* infections or the early escalation of vancomycin-resistant enterococci (VRE) bloodstream infections [61]. The BCID2 panel identifies more bacterial species and relative antimicrobial resistance genes in positive BCs than the BCID panel while retaining similar performance for targets already included in the BCID [62].

Studies evaluating the performance of BioFire^®^ BCID2 showed good agreement with conventional methods for species and antimicrobial resistance identification, especially for monomicrobial cultures, gaining nearly 10 h from time to receive results [63,64]. The main shortcoming identified was the failure to predict third-generation cephalosporin resistance in isolates exhibiting independent cephalosporin resistance mechanisms (4 out of 16 samples containing third-generation cephalosporin-resistant *E. coli*, *Klebsiella oxytoca,* or *K. pneumoniae* isolates were CTX-M negative by BCID2, but carried TEM or SHV, explaining the resistant phenotype) [61], and failure to detect CTX-M ESBL-encoding genes where they were present (one ESBL-producing *Escherichia coli* and two ESBL-producing *Enterobacter cloacae*) [64]. Lower concordance rates for species identification were found in a study analysing central-line-associated bloodstream infections (either detection of more pathogens by the BCID or microbial species misidentified, when compared to VITEK-2) [65]. A recent meta-analysis confirmed the overall good performance of the BCID2 in detecting bloodstream pathogens and associated resistance markers (sensitivity and specificity for gram-positive bacteria on monomicrobial samples were 100% (95% CI 85.8–100%) and 100% (95% CI 90.3–100%), respectively; and for gram-negatives, 100% (95% CI 87.7–100) and 96.9% (95% CI 83.8–99.9%), respectively) [66].

Even though most discordant results between the BCID and blood cultures happen in polymicrobial isolates, interestingly there is the report of a case of polymicrobial bloodstream infection with six organisms identified by the BioFire^®^ BCID that was initially thought to be a monomicrobial infection. Early recognition of specific gram-positive, gram-negative, and fungal organisms and resistance elements allowed for a significantly more rapid optimization of the therapy [67].

Studies evaluating the clinical performance of the BioFire^®^ BCID have been limited by observational study designs and use of historical controls. A randomized trial by Banerjee et al. evaluating primarily antimicrobial therapy duration showed that when BioFire^®^ BCID results were reported with templated comments, there was a reduction in the treatment of contaminants (11% vs. 25% in the control group) and the use of broad-spectrum antimicrobials (12 h reduction in length of use of piperacillin-tazobactam) when compared to standard blood culture bottle processing. With the addition of antimicrobial stewardship, antimicrobial de-escalation was enhanced [68]. MacVane et al. showed the benefits of adding the BioFire^®^ BCID to an established ASP, with shorter times to receive organism identification (17 vs. 57 h in the control group) and earlier de-escalation of antimicrobials when compared to conventional organism identification (48 vs. 63 h in the control group) [69]. However, in both studies, there was no difference in mortality, length of stay in hospital, or cost.

#### 3.3.3. Rapid MBT Sepsityper^®^

Matrix-assisted laser desorption-ionization time-of-flight mass spectrometry (MALDI-TOF MS) has been implemented in clinical laboratories worldwide in the last years, leading to a considerable reduction in turnaround times for pathogen identification and antimicrobial susceptibility.

A 2021 version called the Rapid MBT Sepsityper^®^ IVD kit (Bruker Daltonik, Bremen, Germany) (the term “Rapid Sepsityper Kit” is used to distinguish from the previous Sepsityper kit) allows for an ID of a microorganism by MALDI-TOF MS directly from a positive blood culture in 10 min by skipping the extraction step (subculture on solid media following short-term incubation), with a hands-on time of less than 10 min, with an overall time until identification of 15–20 min after positive blood culture alert [70].

The Rapid Sepsityper^®^ kit was designed to shorten the processing time from ~4 h with the short subculture protocol to ~30 min. It has showed a slightly lower performance when compared to the short subculture protocol, being able to identify 84.5% of 2858 BC gram-negative isolates to the species level, whereas the short subculture protocol identified 90.8% to the species level [71].

The Rapid Sepsityper^®^ protocol combined with MALDI Biotyper^®^ MBT-Sepsityper^®^ module provided a 65.4% and 78.9% reliable identification rate of the species level of monomicrobial positive blood cultures growing gram-positive and gram-negative bacteria, respectively [72].

Other studies found rates of 86.3% and 93.5% for accurately identifying pathogens to the species level in monomicrobial cultures. *Streptococcus pneumoniae*, *Bacteroides fragilis,* and yeasts were the most troublesome to identify [73] and there was poor identification performance for polymicrobial samples using the Rapid Sepsityper^®^ protocol (failure to identify all eight polymicrobial samples) [74].

In a three-site study, the Rapid Sepsityper^®^ protocol enabled the ID of 87.6% of 443 monomicrobial samples (98.6% for gram-negatives, 85.9% for gram-positives, 60.0% for yeasts), and no wrong identification results were observed. Among 55 polymicrobial samples, in 87.3%, one of the two species was identified. For all samples, failed identification involved mainly Coagulase-negative staphylococci (CoNS), yeasts, and *S. pneumoniae* [75]. High non-acceptable identification among *Streptococcus* species was also cited elsewhere [76].

In practical scenarios, time-to-results for the Rapid Sepsityper^®^ protocol can be as fast as 35 min from blood culture positivity [77]. Although the Rapid Sepsityper^®^ protocol enables the rapid identification of pathogens, this method does not currently detect antimicrobial resistance or AMR gene expression [70]. There are no studies to date evaluating the impact on clinical outcomes when using the Rapid Sepsityper^®^ protocol.

## 4. Culture Independent Methods

Detection of pathogens from blood culture samples remains the gold standard for diagnosing blood stream infections [78]. Routine blood cultures may take over 72 h to yield a detectable organism with further time required to identify and test for antibiotic susceptibility.

In this scenario, several culture-independent detection systems have been developed in the last few years [79]. The challenge of identifying pathogens directly from whole blood in septic patients, when pathogen counts can be very low, has led most of these tests to be discontinued.

### 4.1. T2 Magnetic Resonance

T2 magnetic resonance (T2MR) is a diagnostic detection method utilizing miniaturized magnetic resonance technology which measures how water molecules react in the presence of magnetic fields. The T2MR-powered T2Dx^®^ instrument (T2Dx^®^, T2 Biosystems, Lexington, MA, USA) can detect organisms as low as 1 CFU/mL, enabling it to be the only FDA-cleared technology that can detect low levels of pathogens using whole blood [80].

The T2Bacteria panel can detect six clinically relevant bacterial pathogens usually associated with multidrug resistance (*E. faecium*, *S. aureus*, *K. pneumoniae*, *A. baumannii*, *P. aeruginosa*, and *E. coli*), referred to as ESKAPEc pathogens, in 3 to 5 h [81]. Initially, it was not FDA approved for the detection of *Acinetobacter baumannii*, although this organism was already included in the panel. Additionally, after February 2024, the system received FDA clearance for the detection of *A. baumannii* [82]. The panel includes six of the ten most common bacterial species responsible for bloodstream infections along with pathogens with a high probability of being multidrug-resistant organisms (MDROs) [83].

Studies evaluating the clinical performance of the method in detecting the target pathogens in whole blood showed sensitivities ranging from 83.3% to 100% [84,85] and specificities from 90% to 100% [86,87]. However, the sensitivity dropped to as low as 43% for the detection of any bloodstream infection organism in a 2019 study by Nguyen et al. that did not include *Acinetobacter baumannii* in the target organisms [86]. It could, therefore, be recommended as a rapid screening test specifically to detect or rule out the target organisms [84].

A challenging finding from the studies comparing the concordance between the T2Bacteria panel and blood culture was with T2Bacteria-positive/blood culture-negative results [88]. This rate can include either false-negative BC or false-positive T2Bacteria results. In the 2018 work by De Angelis, T2Bacteria-positive/blood culture-negative results were significantly more likely reported among patients receiving antimicrobial therapy [84]. “Infection criteria” have been developed by different groups to try to overcome the low sensitivity of the gold standard blood culture. Patients were classified to the probability of having a BSI, as “Proven BSI”, “Probable BSIs”, “Possible BSI”, and “Unlikely BSI”, according to the results of the T2Bacteria Panel and cultures from the same day from blood and/or cultures from up to 21 days from other sites (such as abdominal fluid, urine, or bronchoalveolar lavage) [84,86,87,89]. This led to some of the T2Bacteria-positive/blood culture-negative results being classified as true positives. Depending on how clinicians interpret the T2Bacteria result, there is potential for increased antibiotic use and, as a result, more *C. difficile* diarrhoea or other complications [90].

The T2MR claims to detect intact cells rather than free-DNA and this might increase the likelihood of detecting microbial cells that are alive and still pathogenic [91]. Culture-independent molecular methods frequently yield additional positive results in culture-negative blood samples [92], many of the discordant results may represent cases of bacterial BSIs that were missed by a culture in the initial paired blood sample.

As a culture-independent method, time to receive results is significantly shorter using the T2Bacteria panel compared to blood culture and can have a mean time from arrival of the sample to the laboratory to species identification of as low as 3.66 h [85]. This allowed for a switch from empirical to targeted therapy within 24 h from sample collection, and patients being prescribed appropriate empirical antimicrobial therapy with a significantly higher frequency among those with a positive T2Dx result when compared to patients for whom T2Dx was not performed [93].

In a recent systematic review by Giannella et al., the use of T2MR was associated with shorter time to receive detection (mean difference (MD) = −81 h; *p* < 0.001) and time to receive species identification (MD = −77 h; *p* < 0.001), shorter time to receive directed therapy (−42 h; *p* < 0.001), and shorter length of hospital stay (MD = −4.8 days; *p* = 0.03) and intensive care unit stay (MD = −5.0 days; *p* = 0.03) [94].

The T2Dx system offers the research use only (RUO) T2Resistance panel (T2R) that detects the following resistance genes within organisms that commonly cause BSIs directly from patient blood samples: KPC, CTXM-14/15, NDM/IMP/VIM, AmpC, OXA, *vanA*, *vanB*, and *mec*A/*mec*C [95]. In a recent prospective observational study for detection of resistance genes in bacterial bloodstream infections, the sensitivity of T2R to detect the following genes in comparison to AST (standard molecular resistance detection systems and phenotypic identification assays) was 100% for CTXM-14/15, NDM/IMP/VIM, AmpC, and *mecA/mecC*, and 87.5% for KPC, directly from the bloodstream within 3 to 5 h from the receipt of the blood sample within the clinical laboratory [96].

### 4.2. Next Generation Sequencing

Next generation sequencing (NGS) of sepsis pathogens is based on the unbiased sequence analysis of circulating cell-free deoxyribonucleic acid (cfDNA) from plasma [97]. cfDNA molecules in circulation originate from dying human cells as well as from colonizing or invasive microbes that release their nucleic acids into the blood as they break down. The technique of microbial cell-free DNA (mcfDNA) sequencing relies on the identification of pathogen DNA fragments (“reads”) released into the circulation from a remote infection site. Detection can be achieved directly from blood; thus, the method has been referred to as a “liquid biopsy” [98]. 

NGS applications for the diagnostics of infectious diseases have demonstrated great potential with successful clinical applications, encompassing the following three distinct approaches: whole-genome sequencing (WGS), targeted NGS (tNGS), and metagenomic NGS (mNGS, also known as clinical metagenomics) [99].

#### 4.2.1. Karius^®^

The Karius^®^ test (KT; Karius, Redwood City, CA, USA) is a laboratory-developed test that detects mcfDNA in plasma. Its performance characteristics were determined by Karius, and it is conducted in the Karius Central laboratory, which is certified according to the Clinical Laboratory Improvement Amendments and accredited from the College of American Pathologists, and so does not require U.S. Food and Drug Administration approval. KT results are available in approximately 24 h from blood sampling [100]. After mcfDNA is extracted and NGS is performed, human reads are removed, and the remaining sequences are aligned to a curated database of >1400 organisms. mcfDNA from organisms present above a statistical threshold are reported and quantified in molecules per microlitre [101].

In the 2017 SEP-SEQ trial, the Karius plasma NGS assay identified a broad range of pathogens in sepsis patients three times more often than blood culture and more frequently than all microbiology tests combined [102]. A subsequent clinical validation of the Karius test showed an overall agreement with blood culture of 93.7% in a cohort of 350 patients in the emergency department with a sepsis alert. It determined sepsis aetiology in 37 additional cases compared to conventional tests [103]. As this test can identify viruses, bacteria, and eukaryotic pathogens, and blood culture is known to have low sensitivity even in sepsis patients, test performance was assessed by comparing the results of microbial cfDNA sequencing with initial blood cultures, all microbiological testing performed within seven days of enrolment (including tissue/fluid cultures, serology and nucleic acid testing) and a composite reference standard consisting of all microbiological test results plus clinical adjudication. This lead to a KT sensitivity of 92.9% with a specificity of 62.7%. The entire clinical picture must be considered when determining the clinical significance of a microbe detected by cfDNA sequencing.

In a cohort of 55 febrile neutropenic patients, KT results compared with BC showed positive and negative agreements of 90% and 31%, respectively; and was twice as likely to provide microbiological diagnosis compared to conventional microbiological tests [104]. In hospitalized adult patients with *Staphylococcus aureus* bacteraemia or gram-negative bacteraemia, the overall sensitivity of KT compared to index blood culture was 89.3%, and the specificity was 74.3% [101]. 

Even though the test has a high sensitivity, current data to define the optimal clinical context for the use of the KT assay are still limited. In a 2021 multicentre retrospective cohort study, KT results led to a positive clinical impact in only 7.3% of patients [105], while a more recent single-centre study showed a positive impact for 30.4% of patients [106]. A 2022 single-centre study demonstrated the clinical impact of KT in solid organ transplant recipients, patients with sepsis, and patients who had been on antimicrobial therapy for less than 7 days, with positive impacts mainly driven by de-escalation of antimicrobial therapy [107]. The test also showed an impact in the discontinuation of antimicrobials [108] and led to a change in antimicrobial management [109]. In the above-cited studies, KT was performed for all indications (e.g., respiratory, central nervous system, joint, invasive fungal infection), not only sepsis. As the KT is based on the fragments of genomic DNA pathogens present in blood, it has been used for non-invasive pathogen detection from plasma even if the infection is at other body sites.

A 2023 systematic review and meta-analysis evaluating the KT in the clinical setting for the diagnosis of infectious diseases, which included five retrospective studies (n = 552) mostly with children, found a pooled positive percent agreement of 67% and pooled negative percent agreement of 70%, when compared to a composite reference standard that included all conventional microbiological testing and clinical history as assessed by an adjudication panel or clinical treatment team, suggesting that it provides limited evidence for ruling in or out the presence of infection [110].

Since September 2023, the Karius test included automatic follow-on testing for seven AMR markers (SCC*mec*, *mecA*, *mecC*, *vanA*, *vanB*, CTX-M, KPC) associated with four classes of antimicrobial resistance across 18 AMR threat pathogens whenever one or more of these pathogens are reported by the Karius test. The results of AMR are reported as an addendum to the original species identification report approximately 24 h later [111].

#### 4.2.2. DISQVER^®^

The DISQVER^®^ pathogen test (Noscendo GmbH, Duisburg, Germany) identifies 1500 described pathogens (bacteria, DNA viruses, fungi, and parasites) within 24 h. The cfDNA in the patient’s blood is determined using NGS. DISQVER’s bioinformatics algorithms compare this information against the Noscendo clinical grade genome reference database. A report detailing all relevant pathogens detected in the sample is provided within a few hours. This knowledge can be used for blood stream infections and sepsis, in oncological diagnostics or diagnostics for endocarditis, for example [112]. DISQVER has an analysis time of 36 to 42 h, including shipping time of up to 24 h [113].

A 2022 study to validate the DISQVER^®^ test for the detection of pathogens in haematologic patients with febrile neutropenia found a 2-fold higher sensitivity (40% for bacteria or fungi) and a broader pathogen spectrum than BC [113]. Another study assessing the diagnostic value of additional NGS pathogen tests in patients with suspected BSI in a surgical ICU showed that NGS may have a higher positivity rate and was able to detect a greater number of pathogens than BC with turnaround times results ranging from one to four days with an average time of 2.5 days [114]. When used in a cohort of 55 patients with heterogeneous diagnoses (23 immunocompromised), the concordance rates of BC and pathogen-specific PCR diagnostics with mNGS testing were 14% (4/28) and 36% (10/28), respectively, and NGS detections would have led to therapeutic consequences in anti-infective therapy for 23 pathogen detections (35% of detections) [115].

## 5. Rapid Antimicrobial Susceptibility Testing

### 5.1. Alfred 60/AST^TM^

The Alfred 60 AST^TM^ (Alifax^®^, Polverara, Italy) system provides antimicrobial susceptibility results directly from positive BCB using light scatter to detect bacterial growth in a liquid culture broth, within 4–6 h. Real-time growth curves of bacteria in the tested antibiotics are compared to growth in a positive control (no antibiotics) and percentage of inhibition of growth (%PIC) is calculated. The %PIC is compared between control and antibiotic tubes and reported as resistant, intermediate, or sensitive categories, according to the range of inhibition [116].

Time taken to obtain antibiogram results of isolated colonies using the Alfred AST^TM^ system can be as short as 3 h to 6.3 h [116,117,118]. This enables clinical decisions to be made within the same work shift in which the episode of bacteraemia is detected.

The Alfred 60AST^TM^ system is unable to provide susceptibility results from polymicrobial samples and anaerobic gram-negative bacteria and relies on gram stain results to set up the correct panel. The antibiotic panel to be tested is composed by the user, which requires prior identification method to assign the proper antimicrobial panel. Another possible drawback is the absence of MIC values and inability of performing susceptibility testing of *Acinetobacter baumannii* or *Stenotrophomonas maltophilia* [116].

This system gives reliable AST results in a short period of time, especially for Enterobacterales and enterococci [118]. Whilst the system provides a notably good performance and high accuracy for gram-negative bacteria, it still needs improvements in gram-positive AST results [116].

CA with conventional methods is above 90% in most studies, with only one study showing a low CA of 62.1% (see Table 2). Differences in the performances in different studies may be due to the choice of antibiotics tested and because strains included in different studies have varying resistance profiles.

Results obtained with Alfred 60AST^TM^ system in gram-negative BSI resulted in earlier discontinuation of aminoglycoside therapy, earlier initiation of effective antibiotic in patients on ineffective empirical antibiotic, shorter times to receive optimal antibiotic, and shorter times to receive escalation of non-aminoglycoside antibiotics [119]. Performance evaluation in a group of 220 patients with a clinical suspicion of severe sepsis or at risk for infections with MDRO led to modification in antibiotic therapy in 37% of all patients [120].

Even with the demonstrated ability to improve AST results compared to conventional methods, no study has yet shown an impact in patient mortality or length of stay.

**Table 2:** Categorical agreement (CA), very major error (VME), major error (ME), and minor error (mE) of Alfred 60/AST (Alifax^®^) system compared to standard of care in different studies.

	Standard of Care	N	CA (%)		VME		ME		mE		Ref.
Barnini Italy, 2016	Vitek2/SensiTitre	1296	62.1		-		-		-		[121]
Giordano Italy, 2018	SensiTitre	405	91.6		14.5%		4.5%		1.2%		[117]
Sánchez-Carrillo Spain, 2019	MicroScan Walkaway	828	97.1		1		22		1		[122]
Boland Belgium, 2019	Phoenix system	679 (109 Gram Pos/570 Gram Neg)	Gram Pos	Gram Neg	Gram Pos	Gram Neg	Gram Pos	Gram Neg	Gram Pos	Gram Neg	[123]
			88.1	92.2	2.3%	0	13.8%	-	12%	-	
Anton-Vazquez London, 2019	Phoenix system	2196 (333 Gram Pos/1863 Gram Neg)	89	95	7	31	29	55	2	5	[116]
Poel Belgium, 2020	Vitek2 or disk diffusion *	595	93.4		8		18		13		[120]
Mantzana Greece, 2021	Vitek2 **	690	94.5		1.45%		3.33%		1.16%		[124]
Cupaiolo Belgium, 2022	Vitek2 or disk diffusion ***	1008	94.9		7.4%		3%		1.8%		[118]
Anton-Vazquez London, 2022	BD Phoenix	709	94		1		7		35		[119]
Curtoni Italy, 2023	Microscan Walkaway	437	94.7		1		10		12		[125]

* Discrepancy analysis was performed by Etest (bioMérieux) or broth microdilution. ** Discrepancies were resolved with MICRONAUT-S (Merlin) or Etest (bioMérieux). *** Discrepancies were investigated using Etest (bioMérieux). Abbreviations: N—number of susceptibility determinations; Gram Pos—gram-positive; Gram Neg—gram-negative. SensiTitre^TM^ (Thermo Fisher Scientific, Waltham, MA, USA), MicroScan (Beckman Coulter, Brea, CA, USA), and the Phoenix system (Becton Dickinson, Franklin Lakes, NJ, USA) are broth microdilution assays.

### 5.2. VITEK^®^ REVEAL^TM^ (Formerly Specific Reveal^®^)

The VITEK^®^ REVEAL^TM^ (bioMérieux) AST system uses a colorimetric sensor technology to detect bacterial populations emissions of volatile organic compounds (VOCs) during growth. It offers extensive antimicrobial coverage for monomicrobial gram-negative bloodstream infections delivering phenotypic AST results in an average of 5.5 h directly from positive blood cultures [126]. It is CE-IVD marked and was very recently (June 2024) FDA cleared [127].

To date, two studies evaluated its performance. Tibbetts et al. found 98.0% EA and 96.3% CA with SensiTitre^TM^, with just 1.3% VME; and 97.0% EA, 96.2% CA, and 1.3% VME *versus* Vitek 2. Organisms tested were *Escherichia coli*, *Klebsiella pneumoniae*, *Klebsiella oxytoca*, *Klebsiella aerogenes*, *Enterobacter cloacae*, *Citrobacter koseri*, and *Pseudomonas aeruginosa*. Average time to receive results (TTRs) for reveal was 4.6 h. Sample preparation was relatively low skill and averaged 3 min [128]. Bianco et al. found an overall CA of 97.6% and EA of 97.7% using positive culture bottles with Enterobacterales, *Pseudomonas aeruginosa*, and *Acinetobacter baumannii* complexes, and analysed 2220 strain/antibiotic pair combinations. Discrepancies were 18 VME (1.6%), 13 ME (1.2%), and 22 mE (2.4%). Reference AST was the MicroScan walkaway automated microdilution system (MicroScan Neg MIC 1 panel). For meropenem/vaborbactam (not included in MicroScan Walkaway), ETEST^®^ (bioMérieux) was used [129].

### 5.3. dRAST^TM^

The QMAC-dRAST^TM^ (QuantaMatrix^®^, Inc., Seoul, Republic of Korea) system provides MIC-based phenotypic antimicrobial susceptibility testing direct from positive blood cultures in 4–6 h from gram staining reports. The system consists of two panels: one gram negative and one gram positive [130].

The positive blood culture sample is directly mixed with agarose and inoculated into a dRAST^TM^ micropatterned plastic microchip consisting of 96 test wells containing various antimicrobials at several concentrations. Bacterial colony formation is detected using time-lapse microscopy. dRAST^TM^ does not require a subculture process nor a precise inoculum size for accurate AST results, and the total time to receive result is less than 24 h [131].

Prospective studies evaluating the clinical performance of the dRAST^TM^ in gram-positive cocci showed a CA of over 94.6% [132,133]. Studies evaluating gram-negatives found a CA of over 92.1% [134,135]. The studies compared dRAST^TM^ and various reference AST methods.

Studies including both gram-positive and gram-negative species found CA above 91.1% [131,136], even when highly resistant organisms were included, although it was not reliable for certain antibiotic-bacteria combinations, such as oxacillin for *Staphylococcus aureus* (VME rate of 7%) and *E. coli* and Piperacillin-tazobactam (VME rate 70.6%) [137,138].

A 2021 randomized controlled trial involving patients with haematological malignancies showed a significant reduction in time to receive results and a higher proportion of patients receiving optimal targeted antibiotics at 72 h with dRAST^TM^ when compared to conventional broth microdilution methods, but was not able to reduce bacteraemia-related mortality rates [139].

### 5.4. ASTar^®^

The ASTar^®^ BC G2 kit (Q-linea AB, Uppsala, Sweden) uses a panel of 23 antimicrobials for the treatment of BSIs caused by gram-negative fastidious and non-fastidious bacteria across a range of 6 to 14 2-fold dilutions, including cefoxitin as a screening agent for AmpC-producing Enterobacterales, testing Enterobacterales, *Pseudomonas aeruginosa*, *Acinetobacter baumannii*, and *Haemophilus influenzae*. It uses time-lapse microscopy of bacterial growth in broth to provide AST results in about 6 h [140].

A performance study with 412 contrived blood cultures and 74 fresh clinical blood cultures, comprising 8650 data points of bacterium-antimicrobial tests found an overall EA of 95.8% and a CA of 97.6% compared to the reference BMD method, with very major discrepancies of 2.4% [141]. Smaller studies found CA and EA of 95.6% and 90.7%, respectively, with 2.4% mE, 2.0% ME, and 2.4% VME [142], and an overall EA of 98% and a CA of 96.1% with an overall rate of ME and VME of 2.5% and 3.3%, respectively [143].

ASTar^®^ was able to correctly identify all patients who required an escalation of antimicrobial therapy and 75% of those who were eligible for de-escalation, but it showed problems with amoxicillin/clavulanic acid CA [141,142]. VME occurred with piperacillin/tazobactam, cephalosporins, and trimethoprim/sulfamethoxazole in *Enterobacteriaceae* and *Pseudomonas* species [142].

### 5.5. Fastinov^®^

FASTinov^®^ is a growth-independent phenotypic technology based on flow cytometry applying fluorescent dyes that reveal bacterial cell damage during treatment. Using specific drugs at breakpoint concentrations, categorical interpretations (susceptible [S], susceptible increased exposure or intermediate [I], susceptible dose-dependent [SDD] or resistant [R]) based on either EUCAST or CLSI criteria are performed by a proprietary software. The FASTinov^®^ report can be released in up to 2 h after a BCB flags positive. The FASTGrampos AST panel covers seven antibiotics (penicillin, ampicillin, cefoxitin, oxacillin, vancomycin, linezolid, and gentamicin) for *Staphylococcus* and *Enterococcus* spp. It includes MIC for Vancomycin for *S. aureus*. The FASTGramneg AST kit covers 12 antibiotics (ampicillin, amoxicilin-clavulanic acid, cefotaxime, ceftazidime cefepime, piperacillin-tazobactam, ceftalozone-tazobactam, ceftazidime-avibactam, meropenem, ciprofloxacin, gentamicin, and amikacin) for *Enterobacterales* spp., *Pseudomonas* spp., and *Acinetobacter* spp. It includes the detection of the main mechanisms of resistance for ESBL and screening for the presence of AmpC and carbapenemases [144].

In the first published study evaluating the potential impact of FASTinov^®^, only gram-negatives were included and the overall agreement between FASTinov^®^ and the reference microdilution method was 98% with a rate of minor, major, and very major discrepancies of 1.03, 1.59, and 1.06%, respectively. FASTinov^®^ R Gramneg kit also provided a correct detection of ESBL-producing strains (CA = 1.00) [145].

Other studies including both gram-positive and gram-negative positive blood cultures (spiked and from hospitalized patients) found CA values for the FASTGramneg panel above 96.4%, and 98.6% for the FASTGrampos panel [146]; and overall Ca of ≥97% and >97% for the FASTGramneg kit and for the gram-positive kits, respectively [147]. Very major errors varied from 0.4% to 0.6%.

## 6. Other Tests and Emerging Technologies

New rapid tests have emerged very recently. As this review focuses on rapid diagnostic innovations, we have included some tests that are not yet FDA/CE-approved and whose clinical performance still needs to be evaluated in practical scenarios.

Molecular methods utilizing PCR for blood stream infections include the CE-IVD marked Molecular Mouse (Alifax^®^, Polverara, Italy), encompassing 64 different targets among gram-positive bacteria, gram-negative bacteria, yeasts, and resistance genes with results in about 1 h from blood cultures [148] and the CE-IVD marked. Unyvero BCU System^TM^ (Curetis GmbH, Holzgerlingen, Germany), which simultaneously tests for 50 targets for pathogen identification and antibiotic resistance markers. Unyvero takes approximately 2 min of hands-on time and provides results in about 5 h from BC positivity [149]. It was recently evaluated for polymicrobial bloodstream infection, where it showed to be a reliable method, despite some limitations concerning detection of anaerobes, Enterococci, and Enterobacterial susceptibility to third generation cephalosporins [150].

Day Zero Diagnostics (Watertown, MA, USA) is developing a genome sequencing-based rapid diagnostic with a machine learning algorithm that identifies both the species and the antibiotic resistance profile of a bacterial pathogen within hours, from bacterial concentrations as low as 1 CFU/mL of blood [151].

With the CE-marked QuickMIC^®^ system (Gradientech, Uppsala, Sweden), microcolonies of gram-negative bacteria are exposed to a linear antibiotic gradient are followed in real time by live imaging. The software analyses the growth patterns of each microcolony along the linear antibiotic gradient, determining antibiotic resistance phenotypically and providing MIC values in 2–4 h direct from positive blood cultures. Bacterial growth can be visually monitored throughout the run for each antibiotic [152].

Many current phenotypic AST methodologies rely on detecting bacterial growth, such as doubling time, emission of volatile organic compounds during growth, or biomass change. This restricts minimizing the TTR. New strategies involve growth-independent phenotypic methods, such as Resistell^TM^ Phenotech (Resistell AG, Muttenz, Switzerland), a nanomotion technology platform to infer viability and response to antibiotics by measuring bacterial vibrations utilising micro-mechanical sensors and machine learning [153]. The oscillation of bacteria at the nanoscale are compared before and after the use of antibiotics, and AST results are available in 2 h from blood culture positivity [154].

FDA-cleared LifeScale (Affinity Biosensors, Santa Barbara, CA, USA) is another growth-independent method for AST that uses a population profiling technology, where a microfluidic sensor counts and measures the masses of individual gram-negative organisms generated for all antibiotics and antibiotic concentrations tested, and an artificial intelligence predictor^TM^ analyses the dataset and provides MIC results in 4.5 h from BC positivity [155].

The Selux NGP system for Rapid AST is an FDA-cleared test, providing phenotypic AST results in 6 h from positive blood cultures. The assay can detect a cell dying without waiting for cell death, by determining the metabolic activity of the cumulative bacterial population using fluorescent markers [156]. In a recent multicentre trial with 2488 isolates (clinical and spiked), including gram-positives and gram-negatives, essential agreement and categorical agreement were >95%, with overall very major error and major error rates of less than 1% [157].

## 7. Host Transcriptomics

Due to the heterogeneous nature of sepsis, accurately detecting it is challenging. The difficulty to distinguish sepsis from non-infectious systemic inflammation is illustrated by the fact that up to 40% of patients receiving antimicrobial treatment in the ICU have no microbiologically confirmed infection [11]. Organ failure typically complicates both sepsis and infection-negative systemic inflammatory disorders, which appear as clinically identical entities [158].

Measuring the host response to infection enables more rapid diagnoses of sepsis than is possible through direct detection of the causative pathogen. Recognizing the time required for pathogen identification in sepsis patients, various investigators have sought to evaluate host immune response differences between septic patients and healthy individuals [159], and also between bacterial and viral infections [160,161,162]. In recent years, a new generation of host immune response diagnostics has been developed based on the analysis of host gene expression through RNA transcripts, referred to as host transcriptomics [163]. Host transcriptomics might be useful as a triage tool to help identify patients who would benefit the most from rapid tests for pathogen ID and antimicrobial susceptibility results.

### Septicyte^®^

The SeptiCyte^®^ technology (Immunexpress, Seattle, WA, USA) is a host response gene expression assay that rapidly quantifies specific biomarkers to aid clinicians in the early identification of infection in suspected sepsis patients. By testing whole blood directly, the assay is able to detect the response of the patient’s immune system to an infection, with a turnaround time of approximately 6 h (from sample draw to report) [164].

SeptiCyte^®^ is a real time PCR-based test for sepsis designed to differentiate non-infectious systemic inflammatory response syndrome (SIRS) from sepsis in critically ill patients, providing an estimate of the likelihood of sepsis (SeptiScore). SeptiCyte^®^ is a CE-marked IVD within the EU, and has FDA 510(k) clearance in the U.S. Its hands on-time is approximately 2 min and results are available in about 60 min [165].

The SeptiCyte^®^ LAB test consists of the simultaneous amplification of four RNA transcripts (CEACAM4, LAMP1, PLAC8, and PLA2G7) in whole blood using a quantitative real-time PCR. Clinical performance studies suggest that SeptiCyte^®^ LAB has a sensitivity above 90% for diagnosing sepsis in critically ill patients, with variable specificity [158].

SeptiCyte^®^ RAPID is a simplified and improved version of the earlier SeptiCyte^®^ LAB test, which achieves the simultaneous amplification and detection of two of the original four RNA transcripts (PLA2G7 and PLAC8) in human blood samples. A recent study for validation of SeptiCyte^®^ RAPID to discriminate sepsis from non-infectious systemic inflammation had areas under the ROC curve (AUCs) ranging from 0.82 to 0.85, a negative predictive value of 0.91 (sensitivity 0.94) for SeptiScore Band 1 (score range 0.1–5.0; lowest risk of sepsis), and a positive predictive value of 0.81 (specificity 0.90) for SeptiScore Band 4 (score range 7.4–15; highest risk of sepsis) [166].

## 8. Discussion

Our rapid review identified seven culture-dependent and two culture-independent assays for pathogen identification in bloodstreams which already have FDA/CE approval (as stated before, the Karius test does not need FDA approval). A further FDA/CE-approved eight commercial assays aim to rapidly predict AST in bacteria causing bloodstream infections.

The performance of the tests was broadly good, being able to correctly identify pathogens and their resistance profiles faster when compared to standard of care. Limitations include the following: problematic identification in polymicrobial samples, ability to identify only on-panel organisms, reliance on gram staining, and poor identification performance for some species.

The inability of most of the rapid tests based on the detection of pathogen genes to universally detect organisms responsible for serious infections represents a limitation and highlights that molecular methods must still currently be used in addition to BC, that retains a crucial diagnostic role. Blood culture is still the most accessible and least expensive way to check for the presence of a pathogen in bloodstream infection.

The same applies to rapid AST, which are only able to detect a limited panel of resistance genes. Also, the presence of a resistance gene does not mean it is being expressed and that the organism will present a resistance phenotype. Some genes (like chromosomal AmpC beta-lactamase in bacteria like Enterobacter) may only be expressed in the presence of antibiotics or by mutations in regulatory genes. As such, the presence of the gene does not necessarily determine the degree of resistance (e.g., to third generation cephalosporins in case of AmpC). Similarly, genes may be detected by molecular methods that are non-functional, leading to false detection of phenotypic resistance. Besides that, not all resistances are single gene/mutation-associated. Resistance can be caused by other mechanisms, such as porin alterations, efflux pumps, target alterations, AmpC de-repression, or multi-gene copies. To date, the available evidence is poor to use antibiotic resistance gene detection to predict phenotypic antimicrobial susceptibility profiles for clinical care [167]. Therefore, phenotypic tests are still needed. Rapid phenotypic susceptibility tests might be a good option. These phenotypic assays may offer information for patient therapy that is safer, more accurate, and more useful. However, a blood culture isolate is still required for phenotypic susceptibility testing.

Polymicrobial bloodstream infections still pose challenges for both genetic and phenotypic rapid AST systems, as well as for rapid pathogen identification, as shown in the lower percentage of agreement with conventional tests described in the previously cited studies. In addition to the speed and agreement with standard of care of rapid tests, their clinical utility is critically linked to timely communication of results to the clinician, which involves a multidisciplinary antimicrobial stewardship program [90].

There have been some advances in rapid culture-independent methods for pathogen detection in bloodstream infections, but their clinical benefit remains unclear, and their implementation has largely failed, given the number of tests discontinued in the last few years. Culture-independent methods for pathogen identification can greatly shorten the time to receive positivity and, like other existing rapid diagnostic methods, when integrated with an ASP may lead to a survival benefit [168].

An important point for current blood culture-independent methods is that they frequently yield additional positive results in culture-negative blood samples [169], leading to the challenge of defining whether the rapid test is a true positive or not, an inherent problem when analysing tests with potentially greater sensitivity than the “gold standard”. In studies that apply clinical adjudication to define true positives of rapid tests, both the sensitivity and specificity of these tests could be overestimated.

NGS from whole blood arises as a promising tool for sepsis diagnosis, with increasing reports of successes. Despite offering hypothesis-free testing with good sensitivity compared to that of multiplex PCR, most next generation sequencing tests still require more than 24 h from sample collection to report results [163], and several hurdles need to be addressed, such as differentiation of DNAaemia from infection, extraneous sources of nucleic acid, method standardization, and data storage, protection, analysis, and interpretation [170].

The high sensitivity of NGS may be attributed to the fact that cfDNA is detectable in blood even during antibiotic treatment [114]. Another reason for the increased diagnostic yield observed in NGS could be the constant presence of bacterial DNA in the bloodstream (DNAemia) even without any signs of infection, as observed by Gosiewski et al. that there is a continuous translocation of bacteria into the bloodstream, which does not always lead to sepsis [171].

As cfDNA can originate from bacterial, fungal, parasitic, and viral microorganisms, NGS can detect multiple pathogens in a single sample, which could be particularly beneficial in patients with suspected polymicrobial infections or unknown focus. But they can also detect organisms of uncertain clinical significance and actionability. Therefore, the results of these tests should be examined with caution and by physicians with ID expertise and familiarity with the technology and result interpretation [103].

Along with BC, other potential applications of NGS include identifying pathogens that cannot be cultured, determining the underlying pathogen in patients presenting with infectious syndromes when standard diagnostics are unable to adequately explain the patient’s clinical presentation or when cultures yield negative results [114].

Methods currently commercially available present restrictions in the process of decision making in sepsis patients, such as the clinical significance of detection in blood and limited evidence of impact on clinical outcomes reported to date (few studies evaluating mortality, length of stay), which justify the current focus on improving culture-dependent methods.

For most of the studies evaluating the tests described in this paper, there are limitations: limited sample size of each individual study, lack of evaluation of clinical impact and results being described mostly as a percentage of agreement or concordance with conventional testing, not reporting diagnostic accuracy measures consistently (e.g., sensitivity, specificity, predictive values, or area under the curve (AUC)), as suggested in standards for reporting diagnostic accuracy studies (STARD) guidelines [172], which also leads to a difficulty in comparing the results between studies.

A limitation in this paper is that we did not apply a standardized quality framework like the QUADAS-2 tool for systematic reviews of diagnostic accuracy studies [173], because this is not a systematic review.

## 9. Conclusions

Rapid tests for pathogen identification, antimicrobial susceptibility testing, and sepsis identification have a great potential for improving the management of patients with bloodstream infections. Culture-independent methods for pathogen identification have emerged in the last few years, but their implementation has not replaced culture; rather, they are an additive to it, but they also add costs without clearly demonstrating benefits to patient outcomes. Rapid antimicrobial susceptibility testing is dependent on blood culture. Most tests are growth-dependent, also suffering from the obstacles imposed by speed of bacterial growth. New technologies aim to contribute to the development of growth-independent methods to obtain faster results. Besides their development, clinical studies are needed to evaluate their real-life performance and impact on clinical outcomes that would justify the additional costs they bring.

## Figures and Tables

**Table 1:** Selected rapid diagnostic tests for pathogen identification in sepsis.

System (Company)	Technology	Regulatory Status (for Bacteria)	Sample	Gram Stain Dependent	ID Targets	Type of AMR Testing	Resistance Markers or Antibiotics Tested
Accelete Pheno^TM^ (Accelerate Diagnostics, Tucson, AZ, USA)	PNA-FISH and morphokinetic cellular analysis	CE marked/FDA cleared	BC	No	6 g-positive 8 g-negative 2 yeasts	Phenotypic	gram-pos: ampicilin, ceftaroline, daptomycin, linezolid, vancomycin, and cefoxitin gram-neg: ampicilin-sulbactam, ciprofloxacin, gentamicin, tobramycin piperacillin-tazobactam, aztreonam cefepime, ceftazidime, ceftriaxone ertapenem, meropenem, and amikacin
Verigene^®^ (Nanosphere, Northbrook, IL, USA)	PCR + microarray	CE marked/FDA cleared	BC	Yes	13 g-positive 9 g-negative	Genotypic	*mecA*, *vanA/B*, CTX-M, KPC, NDM, VIM, IMP, and OXA
cobas^®^ eplex (Roche Diagnostics, Rotkreuz, Switzerland)	PCR + microarray	CE marked/FDA cleared	BC	Yes	20 g-positive 21 g-negative 16 fungal	Genotypic	*mecA*, *mecC*, *vanA*, *vanB* *CTX-M*, *IMP*, *KPC* *NDM*, *VIM* *OXA* (*OXA-23* and *OXA-48*)
BioFire^®^ FilmArray^®^ (bioMérieux, Marcy-l’Étoile, France)	PCR + microarray	CE marked/FDA cleared	BC	No	8 g-positive 11 g-negative 5 Candida species	Genotypic	IMP, KPC, OXA-48-like NDM, VIM, *mcr-1*, ESBL CTX-M, *mec*A/C *mec*A/C and MREJ (MRSA), *van*A/B
Rapid MBT Sepsityper^®^ (Bruker Daltonik, Bremen, Germany)	MALDI-TOF MS	CE marked/FDA cleared	BC	No	>425 organisms (including bacteria and yeasts)	-	-
T2Bacteria Panel (T2Dx^®^, T2 Biosystems, Lexington, MA, USA)	Miniaturized magnetic resonance	CE marked/FDA cleared	WB	No	*E. faecium* *S. aureus* *K. pneumoniae* *A. baumannii* *P. aeruginosa* *E. coli*	-	-
Karius^®^ (Karius^®^, Redwood City, CA, USA)	Metagenomics	- ^¥^	WB	No	>1000 pathogens (bacteria, DNA viruses, fungi and parasites)	-	-
DISQVER^®^ (Noscendo GmbH, Duisburg, Germany)	Metagenomics	CE marked	WB	No	5621 bacteria	-	-

^¥^ Karius is not required to have FDA clearance. Abbreviations: ID—pathogen identification; AMR—antimicrobial resistance; BC—blood culture; WB—whole blood; PNA-FISH—fluorescence in situ hybridization with peptide nucleic acid probes; PCR—polymerase chain reaction; MALDI-TOF MS—matrix-assisted laser desorption-ionization time-of-flight mass spectrometry; CE—European conformity; FDA—U.S. Food and Drug Administration.

## Data Availability

No new data were created or analyzed in this study. Data sharing is not applicable to this article.
